# Air pollution and respiratory health in patients with COPD: should we focus on indoor or outdoor sources?

**DOI:** 10.1136/thorax-2024-221874

**Published:** 2024-10-07

**Authors:** Dimitris Evangelopoulos, Hanbin Zhang, Lia Chatzidiakou, Heather Walton, Klea Katsouyanni, Roderic L Jones, Jennifer K Quint, Benjamin Barratt

**Affiliations:** 1 Environmental Research Group, MRC Centre for Environment and Health, Imperial College London, London, UK; 2 NIHR HPRU in Environmental Exposures and Health, Imperial College London, London, UK; 3 European Centre for Environment and Human Health, University of Exeter, Exeter, UK; 4 Yusuf Hamied Department of Chemistry, University of Cambridge, Cambridge, UK; 5 Department of Hygiene, Epidemiology and Medical Statistics, National and Kapodistrian University of Athens, Medical School, Athens, Greece; 6 National Heart and Lung Institute, Imperial College London, London, UK; 7 School of Public Health, Imperial College London, London, UK

**Keywords:** COPD epidemiology, COPD Exacerbations, Respiratory Measurement

## Abstract

**Introduction:**

While associations between ambient air pollution and respiratory health in chronic obstructive pulmonary disease (COPD) patients are well studied, little is known about individuals’ personal exposure to pollution and associated health effects by source.

**Aim:**

To separate measured total personal exposure into indoor-generated and outdoor-generated pollution and use these improved metrics in health models for establishing more reliable associations with exacerbations and respiratory symptoms.

**Methods:**

We enrolled a panel of 76 patients with COPD and continuously measured their personal exposure to particles and gaseous pollutants and location with portable monitors for 134 days on average. We collected daily health information related to respiratory symptoms through diary cards and peak expiratory flow (PEF). Mixed-effects models were applied to quantify the relationship between total, indoor-generated and outdoor-generated personal exposures to pollutants with exacerbation and symptoms occurrence and PEF.

**Results:**

Exposure to nitrogen dioxide from both indoor and outdoor sources was associated with exacerbations and respiratory symptoms. We observed an increase of 33% (22%–45%), 19% (12%–18%) and 12% (5%–20%) in the odds of exacerbation for an IQR increase in total, indoor-generated and outdoor-generated exposures. For carbon monoxide, health effects were mainly attributed to indoor-generated pollution. While no associations were observed for particulate matter_2.5_ with COPD exacerbations, indoor-generated particles were associated with a significant decrease in PEF.

**Conclusions:**

Indoor-generated and outdoor-generated pollution can deteriorate COPD patients’ health. Policy-makers, physicians and patients with COPD should note the importance of decreasing exposure equally to both source types to decrease risk of exacerbation.

**WHAT IS ALREADY KNOWN ON THIS TOPIC**

- Air pollution is known to worsen respiratory health in patients with chronic obstructive pulmonary disease (COPD). Previous studies have supported these associations using ambient air pollution levels and personal exposure data. It is not yet known whether the health impacts differentiate between indoor and outdoor pollution sources.

**WHAT THIS STUDY ADDS**

- We studied how indoor-generated and outdoor-generated pollutants affect the respiratory health of patients with COPD independent of each other. Pollution from sources within a patient’s home, such as cooking and heating, showed stronger effects on exacerbation than sources from outdoors, with nitrogen dioxide being particularly harmful. Using ambient concentrations as a proxy for exposure, as in the majority of previous studies, can sometimes lead to underestimated health effects.

**HOW THIS STUDY MIGHT AFFECT RESEARCH, PRACTICE OR POLICY**

- Understanding the impacts and regulating daily exposure to both indoor and outdoor pollution is vital for patients with COPD, who can take steps to minimise their exposures, and for caregivers who can offer appropriate guidance. Individuals with respiratory conditions should avoid using gas cookers when possible.

## Introduction

 Exposure to particulate matter (PM) and gaseous air pollutants is an established risk factor for the worsening respiratory health of chronic obstructive pulmonary disease (COPD) patients.^1^ Various metrics have been used for exposure assessment, including ambient concentrations from fixed stations, indoor monitoring at home or personal exposures from portable sensors.^2^,^4^ However, these metrics are influenced to varying degrees by both indoor (eg, cooking) and outdoor (eg, traffic) sources of pollution. For example, the same molecule, indoor-generated nitrogen dioxide (NO_2_) is coemitted with a different mixture of chemicals than outdoor-generated NO_2_ with potentially different health impacts. This distinction is particularly important for PM, as the chemical composition (and therefore the relative toxicity) depends strongly on the source.

These differences cannot be captured directly with air quality monitors and consequently introduce two main barriers to effective COPD management concerning environmental exposures by patients and practitioners. First, despite the distinct physical and chemical characteristics of the indoor and outdoor air, little is known about potential source-related health effects. Second, the methods to mitigate exposure to individual air pollution sources are very different and, in some cases, conflicting.

Novel analytical methods are needed to disaggregate the indoor-generated and outdoor-generated components of exposure. Few previous studies have separated indoor-generated (PeIG) and outdoor-generated personal exposures (PeOG),^5 6^ and even fewer have assessed the potential health effect differentiation for indoor and outdoor sources.^7 8^ Total personal exposure to PM or gases, such as NO_2_ or ozone (O_3_), as measured by portable monitors has been considered as the ‘gold-standard’ exposure estimate in epidemiological studies^4^ and in assessing measurement error.^9^ However, this metric is unable to differentiate the health effects of indoor and outdoor pollution and is not directly comparable with the ambient pollution levels.^10^

Within the ‘Characterisation of COPD exacerbations using environmental exposure modelling’ (COPE) project, we previously demonstrated that it is feasible to collect personal air pollution measurements for multiple pollutants that have been associated with adverse health effects on patients with COPD,^1^ for extended time periods.^11^ We found statistically significant associations between total personal exposure to gaseous pollutants and COPD patients’ respiratory health but not for PM.^12^ However, we did not, at the time, assess whether these associations were driven by indoor-generated or outdoor-generated pollution for gases, or if the lack of association with particles was a consequence of using total personal exposure, masking the effects of specific sources. Identifying and prioritising specific harmful sources of pollution for people’s health is crucial for patients, clinicians and policy-makers to improve the quality of life of patients and public health.

In light of these concerns, we present, to our knowledge, the largest respiratory health study to date that separates total personal exposure to PM_2.5_, NO_2_, O_3_ and carbon monoxide (CO) into indoor-generated and outdoor-generated pollution. We assessed whether the disaggregation leads to refined health estimates compared with widely used exposure metrics in air pollution epidemiology, that is, total personal or ambient exposure estimates. To do that, we associated the acute respiratory health outcomes of 76 patients with COPD with improved exposure metrics extracted from personal and ambient exposure measurements over an average of 134 days each.

## Methods

The COPE study incorporated a large exposure measurement campaign within the Greater London area. Details about the study are available elsewhere.^11 13^ Briefly, we followed up 130 ex-smoking patients with COPD from May 2015 to October 2017, and for 76 of whom, we performed the personal exposure source separation; 54 participants who provided data for less than a month or lived primarily outside Greater London were excluded. Baseline information was collected with a general questionnaire on recruitment and training was provided to each participant by a research physiotherapist in the use of a portable peak flow metre (PFM) and diary cards for recording daily respiratory symptoms. No participants were current smokers or held jobs that could be characterised as having significant occupational exposure.

### Exposure assessment

A validated personal air quality monitor was used to collect continuous measurements of PM_2.5_, NO_2_, CO, O_3_ and GPS coordinates at 1 min resolution. The monitors showed good reproducibility and agreement with reference monitor in different microenvironments with mean R^2^>0.8.^14^ Spatial analysis of GPS data was performed to tag each minute of data into ‘home’, ‘other indoor’ (other than home) and ‘outdoor/transit’.^15^ Air pollution concentrations outdoors at each home address were estimated using concurrent measurements from ambient monitoring stations in London, scaled by the London Air Quality Toolkit (LAQT) modelled estimates.^16^ Instead of the nearest monitor to each participant’s residence, we used a matched monitor approach informed by the LAQT model to better approximate the background residential outdoor air pollution (online supplemental material).

Indoor air quality is affected by outdoor air pollution infiltrating inside, modified by indoor sources, chemical sinks or deposition processes. When participants were at home, the outdoor-generated component of exposure (PeOG) that infiltrated indoors was estimated with an empirical model described previously, which is able to separate out data points unaffected by indoor sources.^17^ These separated data points, together with corresponding ambient measurements, were used to calculate an infiltration efficiency for each home, then applied to ambient data to estimated exposure to outdoor sources (PeOG). The indoor-generated component of exposure (PeIG) was then estimated by subtracting PeOG from the total exposure measured with the personal monitors (PeT). When participants were outdoors or in other microenvironments, we assumed no other indoor sources and the measured total personal exposure was regarded as personal exposure from outdoor sources. More details on dataset preparation, matching with ambient data and the partition of indoor-generated and outdoor-generated pollution, can be found in online supplemental material.

### Health outcomes

Participants filled in their diary cards every evening, indicating the occurrence of symptoms, medication use and disrupted sleep patterns. A respiratory clinician verified the diary cards and defined an exacerbation as a sustained worsening of symptoms for at least 2 days beyond normal variation.^18^ Participants measured their daily peak expiratory flow (PEF) using a PFM at the same time every day.

### Statistical analysis

Exposure variables were aggregated to daily means, except for ozone, for which we calculated a daily 8 hours maximum. Mixed-effects logistic regression models were applied with a random intercept per participant for the occurrence of exacerbation and daily symptoms. For PEF, we applied linear random intercept models. Each model was fitted four times, one for each exposure variable of interest, that is, total, PeIG and PeOG, and ambient concentrations. The associations with total personal exposure have been assessed in our previous publication, but we include them here for comparability with the other exposure metrics. All models were adjusted for a predefined set of potential confounders, including age, sex, COPD severity defined by airflow obstruction using spirometry, medication records and the Global Initiative for Chronic Obstructive Lung Disease (GOLD) classification,^11^ socioeconomic status (SES), medication use on the day, temperature measured from the personal monitor and time trends. For SES, we used the Index of Multiple Deprivation score at postcode level, temperature and time were adjusted using natural spline functions. Potential prolonged effects were investigated by introducing lagged exposures up to day three and the average lag 0–3. Model estimates were reported as per IQR increase in each exposure. The IQR increase provides a plausible exposure increment and assists comparability between epidemiological estimates for exposures of different scale. Exposure–response relationships per 1 unit increase were also quantified (0.1 ppm for CO). STATA V.16 and R V.4.1.2 were used.

## Results

### Descriptive statistics

We performed the exposure separation on 76 out of 130 participants, with an average/median of 134/150 person-days and a mean age at recruitment of 71 years, of whom 39 (51%) were females, 55 (72%) used inhaled corticosteroids and 29 (38%) had severe or very severe COPD (table 1). For the respiratory outcomes, exacerbation was reported in 13% of the person-days, the rarest symptom was sputum (7%) and the most frequent was breathlessness (16%). The average PEF measurement was 236 L/min. Patients stayed at home all day for 50% of the person-days, and their mean time spent outdoors daily was 1.5 hours. Median time spent in other indoor environments was 0 hours, with a mean (SD) of 0.02 (0.1) hours (results are not shown).

**Table 1:** Descriptive statistics for health outcomes and confounders included in the analysis (baseline characteristics and repeated measurements)

Variable	Mean (SD)* or n (%)†
**Baselinecharacteristics (76 participants)**	
Sex (females)—n (%)	39 (51.3)
Age—mean (SD)	70.7 (7.8)
Medication use: inhaled corticosteroids (yes)—n (%)	55 (72.4)
Index of Multiple Deprivation Score—mean (SD)	21.1 (12.3)
COPD severity—n (%)	
Mild	12 (15.8)
Moderate	35 (46.1)
Severe	19 (25.0)
Very severe	10 (13.2)
**Repeatedmeasurements (10 210person-days)**	
Exacerbation (yes)—n (%)	1363 (13.4)
Breathlessness (yes)—n (%)	1608 (15.8)
Cough (yes)—n (%)	1467 (14.4)
Sleep disturbance (yes)—n (%)	1042 (10.2)
Sputum (yes)—n (%)	710 (7.0)
Wheeze (yes)—n (%)	1146 (11.2)
Peak expiratory flow (L/min)—mean (SD)	236 (105)
Ambient temperature (^o^C)—mean (SD)	21.1 (2.4)
Time spent outdoors per day (hours)—mean (SD)	1.5 (1.2)
Stayed all day home (yes)—n (%)	5113 (50.1)

* Mean and Standard Deviation (SD)SD for continuous variables.

† Number of occurrences (n) and percentage across the whole sample/person-days for categorical variables.

COPDchronic obstructive pulmonary disease

The daily mean (SD) total personal exposures were 7.9 (6.1) ppb for NO_2_, 12.1 (13.8) µg/m³ for PM_2.5_, 0.12 (0.10) ppm for CO and 6.4 (4.7) ppb for O_3_. The corresponding ambient measurements were considerably higher than total personal exposure for the gaseous pollutants but were similar for PM_2.5_ (table 2). Mean personal exposure from indoor sources was similar to outdoor for NO_2_ and CO, higher for PM_2.5_ and lower for O_3_. The NO_2_ and PM_2.5_ SD were higher for PeIG compared with PeOG.

**Table 2:** Descriptive statistics for the exposure data included in the analysis (repeated measurements for 76 COPE participants)

Exposure variable		Person-days	Mean (SD)	Min.	25th percentile	Median (IQR)	75th percentile	Max.
NO_2_ (ppb)	PeT	10 210	7.9 (6.1)	0.3	4.3	6.6 (5.5)	9.7	76.5
	PeIG	10 116	4.0 (5.2)	0.0	1.4	2.6 (3.5)	4.9	71.8
	PeOG	10 116	4.0 (2.9)	0.2	2.2	3.3 (2.8)	5.0	69.8
	Ambient	9995	16.9 (8.9)	1.0	10.5	15.6 (11.1)	21.5	69.2
PM_2.5_ (µg/m**³**)	PeT	9636	12.1 (13.8)	0.1	5.3	8.4 (8.7)	14.0	278.2
	PeIG	9375	6.8 (12.2)	0.0	1.7	3.8 (5.9)	7.6	267.9
	PeOG	9369	5.5 (8.2)	0.0	2.1	3.4 (3.8)	6.0	378.4
	Ambient	9609	13.2 (10.7)	2.3	6.7	9.5 (8.7)	15.4	122.0
CO (ppm)	PeT	10 209	0.12 (0.10)	0.0	0.1	0.1 (0.08)	0.1	4.1
	PeIG	10 174	0.06 (0.06)	0.0	0.0	0.0 (0.06)	0.1	0.9
	PeOG	10 174	0.06 (0.06)	0.0	0.0	0.1 (0.03)	0.1	3.5
	Ambient	10 137	0.19 (0.13)	0.1	0.1	0.2 (0.09)	0.2	2.0
O_3_ (ppb)	PeT	10 210	6.4 (4.7)	1.0	3.3	5.2 (4.8)	8.1	57.5
	PeIG	9828	2.8 (3.2)	0.0	1.0	2.0 (2.7)	3.6	54.5
	PeOG	9828	4.4 (3.6)	0.0	2.2	3.5 (3.3)	5.5	51.6
	Ambient	9269	32.8 (13.1)	0.4	25.3	33.8 (15.4)	40.7	95.6

COPDchronic obstructive pulmonary diseaseCOPEcharacterisation of COPD exacerbations using environmental exposure modellingPeIGpersonal exposure to indoor generated pollutionPeOGpersonal exposure to outdoor generated pollutionPeTtotal personal exposure

We observed strong within-person Pearson correlation coefficients (R) between total and indoor-generated personal exposure for all pollutants (R 0.72–0.86), with weaker correlations for outdoor-generated (R 0.51–0.64) for all pollutants except ozone (R 0.73). The correlations between ambient levels of the four pollutants were considerably higher than those between the other exposure metrics (online supplemental material). This indicates that variation in total personal exposure is driven by variation in both indoor and ambient sources. In contrast to what is frequently observed in epidemiological studies using ambient measurements, within-person correlations between pollutants were relatively low. This can be attributed to the high variability of indoor sources operating in different microenvironments compared with outdoors where sources have relatively small variation.

### Epidemiological findings

**Figure 1:**
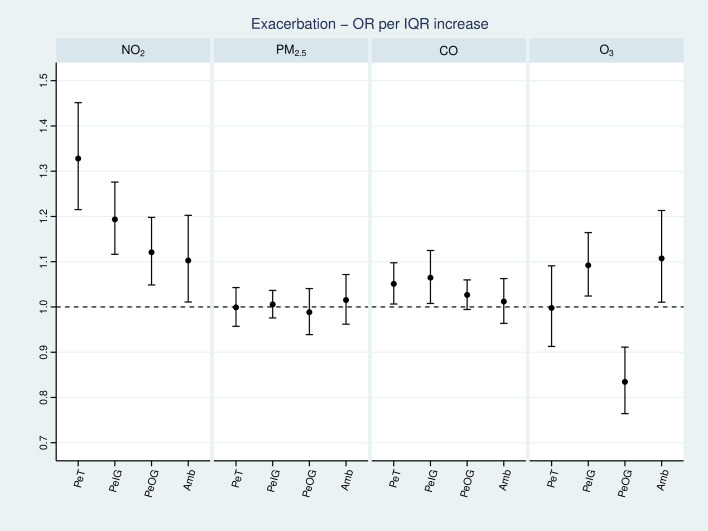
OR with 95% CI for the occurrence of exacerbation associated with an IQR increase on the same day (lag0) for each air pollutant and exposure variable. Random intercept models adjusted for age, sex, COPD severity, Index of Multiple Deprivation Score, inhaled corticosteroids medication use, temperature and time. COPD, chronic obstructive pulmonary disease; NO_2_, nitrogen dioxide; PeIG, personal exposure to indoor generated pollution; PeOG, personal exposure to outdoor generated pollution.

We observed a strong relationship between total personal exposure to NO_2_ and COPD exacerbation, with the odds of occurrence increased by 33% (95% CI (22%, 45%)) for an IQR increase in NO_2_ (figure 1). This was driven by both indoor (OR 1.19 (1.11, 1.28)) and outdoor (OR 1.12 (1.05, 1.20)) sources. Ambient levels of NO_2_ were found to have a smaller association (1.10 (1.01, 1.20)). For CO, we found marginally statistically significant associations for total personal exposure (1.05 (1.01,1.10)), driven by indoor sources (1.06 (1.01, 1.12)). Total personal exposure to ozone had no overall effect on COPD exacerbation (1.00 (0.91, 1.09)), although exposure levels were very low (mean 6.4ppb) in comparison with ambient (32.8ppb), due to its reactivity indoors. Both ambient and indoor-generated ozone had a harmful effect on exacerbations (1.11 (1.01. 1.21) and 1.09 (1.02, 1.16) respectively), while a protective effect of outdoor-generated ozone was observed (0.83 (0.76, 0.91)). This might be attributed to increased ventilation rates that would elevate indoor levels of PeOG O_3_. We observed no association with particles for any of the exposure variables under investigation indicating that our previous findings were robust even when using advanced exposure metrics.^12^

**Figure 2:**
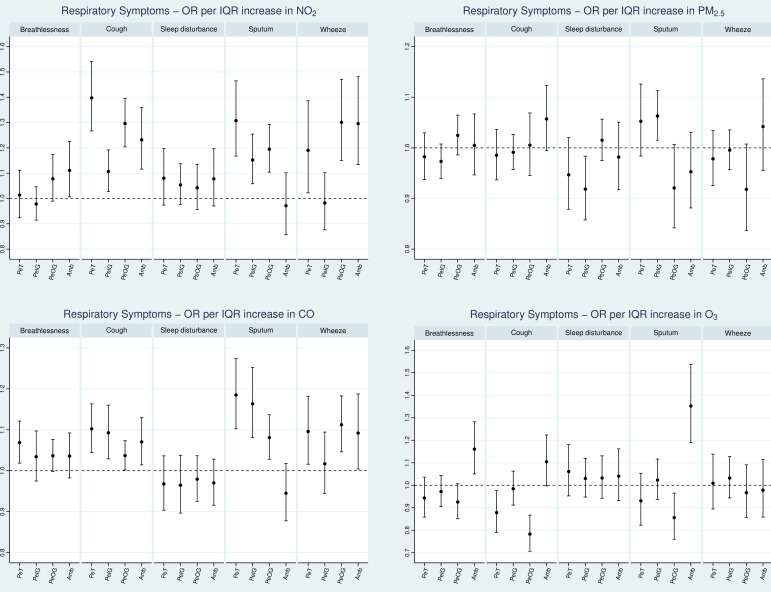
OR with 95% CI for the occurrence of respiratory symptoms associated with an IQR increase on the same day (lag0) for each air pollutant and exposure variable. Random intercept models adjusted for age, sex, COPD severity, Index of Multiple Deprivation rank, inhaled corticosteroids medication use, temperature and time. COPD, chronic obstructive pulmonary disease; NO_2_, nitrogen dioxide; PeIG, personal exposure to indoor generated pollution; PeOG, personal exposure to outdoor generated pollution.

We also investigated associations between five individual respiratory symptoms and exposure variables (figure 2). NO_2_ was again found to have the highest health effect estimates per IQR increase, with statistically significant estimates observed for all outcomes, except for sleep disturbance. Interestingly, outdoor-generated NO_2_ often had higher ORs compared with indoor-generated. Also, PeOG resulted in higher effect estimates compared with using ambient concentrations as an exposure proxy for all respiratory symptoms except breathlessness.

CO exposure was statistically significantly associated with all health outcomes except sleep disturbance. In contrast to NO_2_, PeIG CO had higher effect estimates than PeOG CO except for wheeze. For O_3_, there is no general pattern, as both positive and negative associations were observed but most of them were not statistically significant. Exposure to particles does not seem to have an association with any respiratory symptom.

In general, considerable variation was observed in the estimates of PeIG and PeOG, especially for NO_2_ and O_3_ (probably because of their higher chemical reactivity) and to a lesser extent for PM_2.5_ and CO. The estimates for total personal exposure seem to additively combine those for indoor-generated and outdoor-generated exposure. We identified cases in which using total personal as the exposure variable expresses the total effects of a pollutant as a chemical, combining the indoor-generated and outdoor-generated personal exposure estimates. However, ozone demonstrated more complex relationships, likely reflecting the strong inverse correlation with NO_2_ in ambient air observed in numerous epidemiological studies.^19^

Finally, we explored the relationships between changes in PEF and total personal exposure, PeIG, PeOG and ambient levels of pollution (table 3). Total personal exposure to PM_2.5_ was associated with a decrease in PEF (−0.53 L/min (−0.83, –0.23)), which was mainly driven by PeIG (−0.31 (−0.54, –0.09)). Both effect estimates remained significant for those who spent all day at home. The findings for the gaseous pollutants were not statistically significant, except for a marginal positive association between total NO_2_ exposure in all participants (0.58 L/min (0.04, 1.12)) and PeOG NO_2_ in those who stayed all day at home (1.74 (0.75, 2.73)).

**Table 3:** Average change in peak expiratory flow (PEF) and 95% CI associated with an IQR increase on the same day exposure

Pollutant	Exposure variable	Average change in PEF (L/min) per IQR increase (95% CI)		
		All person-days	All-day at home	Not all-day at home
PM_2.5_	PeT	**−0.53 (−0.83,0.23)**	**−0.56 (−0.94,0.17)**	**−0.50 (−0.93, –0.06)**
	PeIG	**−0.31 (−0.54,0.09)**	**−0.43 (−0.70,0.15)**	−0.04 (−0.41, 0.32)
	PeOG	−0.16 (−0.39, 0.06)	0.051 (−0.60, 0.71)	−0.22 (−0.46, 0.01)
	Ambient	−0.07 (−0.45, 0.30)	0.15 (−0.39, 0.69)	−0.27 (−0.76, 0.23)
NO_2_	PeT	**0.58 (0.04, 1.12**)	0.54 (−0.16, 1.25)	0.57 (−0.08, 1.22)
	PeIG	0.14 (−0.24, 0.53)	0.06 (−0.44, 0.55)	0.37 (−0.13, 0.87)
	PeOG	0.37 (−0.09, 0.83)	**1.74 (0.75, 2.73**)	−0.10 (−0.61, 0.41)
	Ambient	0.20 (−0.41, 0.81)	−0.25 (−1.13, 0.63)	0.46 (−0.30, 1.23)
CO	PeT	−0.05 (−0.40, 0.31)	−0.18 (−0.68, 0.33)	0.06 (−0.41, 0.54)
	PeIG	0.14 (−0.28, 0.56)	0.39 (−0.17, 0.94)	−0.00 (−0.60, 0.60)
	PeOG	0.19 (−0.06, 0.45)	0.43 (−0.21, 1.07)	0.10 (−0.17, 0.38)
	Ambient	−0.20 (−0.55, 0.14)	−0.16 (−0.65, 0.32)	−0.24 (−0.69, 0.20)
O**_3_**	PeT	−0.35 (−0.91, 0.21)	−0.45 (−1.16, 0.26)	−0.30 (−1.02, 0.43)
	PeIG	−0.21 (−0.62, 0.21)	−0.17 (−0.70, 0.36)	−0.16 (−0.75, 0.43)
	PeOG	−0.05 (−0.57, 0.47)	−0.73 (−1.75, 0.29)	−0.04 (−0.60, 0.52)
	Ambient	0.31 (−0.28, 0.90)	0.41 (−0.42, 1.23)	0.24 (−0.53, 1.01)

Results were also presented for those who spent all day at home or not. Random intercept models adjusted for age, sex, COPD severity, Index of Multiple Deprivation rank, inhaled corticosteroids medication use, temperature and time. In bold are the statistically significant estimates.

COPDchronic obstructive pulmonary diseasePeIGpersonal exposure to indoor generated pollutionPeOGpersonal exposure to outdoor generated pollutionPeTtotal personal exposure

### Sensitivity analysis

The findings remained consistent when we adjusted PeIG for PeOG and vice versa, or for another pollutant from the same source, for example, PeOG PM_2.5_ adjusted for PeOG NO_2_ (online supplemental material). Interestingly, the only significant changes were found in the effect estimates of PeIG CO or O_3_ when adjusted for PeIG NO_2_. For PeIG CO in particular, the adjusted estimate reduced substantially and became not statistically significant. This suggests that the CO estimate may reflect the effects of the pollutant mixture from indoor combustion, rather than pollutant-specific effects, especially if one takes into account the low CO exposures observed in our study. The effect estimates for PeIG O_3_ increased when adjusted for PeOG O_3_, that is, from 1.09 (1.02, 1.16) to 1.12 (1.05, 1.19).

When we assessed whether there is an effect modification of the air pollution–exacerbation relationship by staying all day at home or not, we found considerably higher regression estimates for those who spent some time outside compared with those who did not for all exposure variables except for ambient ozone (online supplemental material). This was expected for PeOG, but not for PeIG as overall IQRs for the whole population were used as exposure increments. This may imply that people adjust their lifestyle, and, thus, their exposures when they experience an exacerbation or other respiratory symptoms. However, when we assessed the effects of lagged exposures up to 3 days before the occurrence of exacerbation, we found similar and, in many cases, higher effect estimates compared with the same day exposure (online supplemental material). The lag0-3 estimates were even higher indicating prolonged, cumulative effects mainly for gaseous pollutants but also for indoor-generated particles.

We also estimated the associations between all the exposures and outcomes under investigation per 1 unit exposure increment (0.1 ppm for CO), instead of per IQR increase (online supplemental material). The OR for exacerbation occurrence associated with 1 ppb increase in PeT and PeIG were similar, that is, 1.053 (1.036, 1.070) and 1.052 (1.032, 1.072), respectively and slightly higher than PeOG, that is, 1.041 (1.017, 1.066). However, a fixed exposure increase, such as 1 ppb, for different exposure metrics, may not be directly comparable, as one unit increase in total personal exposure might be similar to a fraction of that for PeIG and PeOG.

## Discussion

We investigated associations between personal exposures to multiple pollutants from indoor and outdoor origins with the respiratory health of 76 people with COPD over an extended period (up to 6 months). To our knowledge, this is the first study that assessed the potential differentiation of the acute health effects of personal exposure from different sources on such a large scale. We found higher health effect estimates per IQR increase in the corresponding pollutant for indoor-generated pollution and exacerbation compared with outdoor-generated pollution, although both source types were significant. Using the ambient concentrations which may be an error-prone exposure, resulted in health effect attenuation, especially for NO_2_, thus, this pollutant might be more harmful than previously thought. Findings from studies that use this type of exposure metric should be interpreted with caution due to potential measurement error bias towards the null.

Identifying the most harmful sources of pollution in people’s personal exposure is important for policy-makers, physicians and patients with COPDs. In most regions, policy-makers currently focus almost entirely on improving ambient pollution levels through specific measures and regulations, but little attention is paid to indoor-generated pollution. However, for patients with COPDs who tend to be older and stay more time indoors, indoor-generated air pollution, either by gas cookers or heaters that are more related to NO_2_ exposure, or by smoking and fireplaces that contribute to PM exposure, may be of more importance compared with other population groups. Awareness of the major sources of air pollution allows respiratory physicians to provide personalised environmental medicine to their patients aiming to directly and immediately decrease exacerbations or other symptoms. Not only will this improve COPD patients’ health and quality of life, but also it will reduce the burden on the healthcare system with associated health impact reduction and monetary benefits. Raising awareness of the harmful sources of air pollution for patients with COPD can lead to behavioural change that will reduce their exposures and, as a result, improve their respiratory health. While awareness of outdoor sources of air pollution, especially traffic, is improving in many countries, awareness of indoor sources remains low. Our study results show that a focus on indoor combustion sources, such as gas stoves and heaters, and the potential elimination of these sources would have even greater benefits to patients.

We found that the largest effect estimates were linked to indoor generated NO_2_, which also displays a larger variability compared with outdoor generated NO_2_ exposure. The dominant source of NO_2_ indoors is from gas cooking and, to a lesser extent, heating.^20^ This finding aligns with established relationships between respiratory health and the use of gas cookers, especially in asthmatics.^21^ There is a move in European countries and some US states to legislate against installing gas into new build homes. While this initiative is based on carbon emission reduction, it would also almost entirely remove indoor generated NO_2_ and CO from the home environment, leading to potentially significant health gains among patients with COPD and the general population.

Only a small number of studies have tried to separate outdoor-generated and indoor-generated pollution and quantify the associated health effects of each source. Four studies have been identified, although with small sample sizes. Ebelt *et al*^7^ used time-activity data and sulphate measurements as a tracer of PM infiltration, to estimate ambient and non-ambient exposures to PM for 16 patients with COPD (104 person-days).^7^ They assessed respiratory and cardiovascular endpoints and found no associations with total and non-ambient personal exposures. They found some statistically significant associations with PeOG which were generally equal to or larger than those for the ambient concentrations. Koenig *et al*^22^ and Allen *et al*^23^ found in the same sample that outdoor-generated particles are associated with increases in airway inflammation (exhaled nitric oxide).^22 23^ A more recent study from Ni *et al*^8^ estimated outdoor-originated personal PM exposure and its association with lung function for 33 patients with COPD (170 person-days).^8^ They found no association between PEF and either PeOG or ambient concentrations. However, for other lung function measurements, they found higher effect estimates for an IQR increase in ambient levels compared with PeOG, but this might be due to the large difference in scale between PeOG and ambient levels (IQRs of 45.3 and 111 µg/m^3^, respectively). We had similar findings for ozone, which is a pollutant that reacts indoors, and thus, PeOG is low, especially compared with ambient concentrations. However, both studies included only a small number person-days (n=104 and 170) in comparison with our study (n=10 184). Additionally, the chemical composition and potential toxicity of indoor-generated PM between countries might be highly heterogeneous due to the diverse sources operating in distant settings.

Our analysis required extensive processing of large volumes of personal measurement data to separate indoor from outdoor sources. Personal or wearable pollution monitoring sensors may not be as accurate as reference monitors.^14^ Therefore, reductions in measurement error achieved by using personal assessments of exposure may have been countered by increases in instrument error, but our personal measurements showed good agreement with standard instrumentation in indoor, outdoor and mobile deployments in the UK.^14 15^ We also showed that the error introduced from the instrument uncertainty was larger than the error introduced when using ambient measurements as metrics of exposure.^14^ Errors might have been introduced most likely in the case of indoor O_3_, which might have been insignificant as levels were very low and close to the limit of detection of the sensors. Moreover, further improvements in source identification, such as the inclusion of indoor environments other than the home through location tagging, will add new insight and relevance of application to population subgroups who do not spend such a large proportion of their time at home. For PM specifically, we could not separate the constituents of PM exposure which would have provided further insight on the differentiation of the effects of combustion-derived and non-combustion-derived particles. Future research could use robust source apportionment techniques in exposure assessment to identify exposures to the most toxic components in the PM mixture and indoor volatile organic compounds (VOCs) which is currently almost completely lacking and could enable efficient environmental policy.

Ambient levels of the pollutants were found to be more correlated with each other, compared with any personal exposure variable (total, indoor-generated and outdoor-generated—online supplemental material). This is extremely important for two-pollutant or multipollutant epidemiological modelling through which one can disentangle the independent effects of the pollutants accounting for potential confounding from the other pollutants. High correlations between the exposures create health effect estimates that are unstable and difficult to interpret. Low correlation between personal exposures was probably the reason for not observing large changes in our two-pollutant models, except for O_3_ or CO adjusted for NO_2_. For O_3_, this was expected due to the interlinked formation of these pollutants and its negative correlation with NO_2_, but for CO it may be an indicator of the NO_2_ effect and their common indoor combustion sources (such as gas cooking). As the levels of CO were very low in comparison with ambient health guidelines, in this study, CO might be a proxy for other coemitted pollutants.

## Conclusions

Regulating day-to-day exposure to both indoor and outdoor sources of gaseous pollution is important to the respiratory health of patients with COPD in London. There are actions that patients can take to reduce these exposures as well as legislative interventions. Those caring for patients with COPD should be aware of these actions and provide appropriate advice. Those with respiratory conditions should avoid the use of gas cookers where possible.

## supplementary material

10.1136/thorax-2024-221874 online supplemental file 1

## Data Availability

No data are available.
