# Supplementary material for: Air pollution and respiratory health in patients with COPD: should we focus on indoor or outdoor sources?
Source: Thorax. 2024 Oct 7;79(12):e221874. doi: 10.1136/thorax-2024-221874 (PMC11671933; doi:10.1136/thorax-2024-221874)
Supplement: online supplemental file 1 [file thorax-79-12-s001.pdf]

Supplementary material

Exposure separation methodology:

The partition of total personal exposure into indoor- and outdoor-generated pollution for this study is based on the methods described in our previous publication (Zhang et al. 2022) and will be soon published in a separate publication. Briefly:

The participants were provided with a personal air quality monitor (PAM) designed for this study. The portable monitor measured gaseous pollutants (NO_2, O_3 and CO) and particles ($PM_{2.5}$) at 1-minute resolution. Measurements were fully ratified following a QA/QC protocol (Moore et al. 2016). The PAM also recorded GPS position. Only home locations in Greater London and participants that provided data for more than one month were included in the final dataset for the exposure separation.

Chatzidiakou et al. (2022) classified the location of the participants into microenvironments including 'home', 'other indoor', and 'outdoor/transit', using 1-min resolution GPS data. Location data was combined with daily diary data. Location was set as missing if the participants left the home without taking the monitor with them during daytime, when they were on holidays, and when instruments had sensor failures. From the 1-min resolution tagged data, we estimated 15-min resolution air pollution personal averages and the location assigned was the one in which the participant spent most of the 15-minute time period.

In order to estimate ambient pollution data for each participant's home address at 15-min resolution, we used measurements from fixed-site reference monitoring stations of the London Air Quality Network (www.londonair.org.uk), and estimates from the London Air Quality Toolkit (LAQT) model (Kelly et al. 2011). We used the LAQT annual model results at 20x20m grid that included the participant's home location and covered the majority of participant's measurement period. This annual modelled estimate was compared with the LAQN data for each monitor in London and matched with the monitor that had the closest annual estimate, instead of the nearest one to the participant's residence, which might not adequately capture the more representative urban/regional background due to strong local (to the station) sources. Each participant was assigned the 15-min measurements from the matched monitor, scaled by the ratio between the annual levels of the LAQT modelled estimate and the LAQN matched monitor annual average. Personal exposure measurements recorded at home and the matched ambient data were used as inputs for infiltration efficiency calculation. Only indoor data points when no indoor source event occurred were included into infiltration efficiency calculation. Such points were identified during a series of censoring stages. For example, if one indoor $PM_{2.5}$ data point is 1.5 times and 4 $\mu g/m^3$ higher than the previous measurement and also higher than the corresponding outdoor $PM_{2.5}$ at the same time, while outdoor $PM_{2.5}$ data point is not 1.5 times higher than the previous measurement, this data point is identified as the start of rising edge of the indoor source event. After all indoor data points affected by indoor source event were identified, the remaining indoor data points and corresponding ambient pollution data were included into a multiple linear regression model to calculate infiltration efficiency according to a mass balance model (Allen et al., 2003; Zhang et al., 2022). For other pollutants, the same procedure applies but with adjusted thresholds for each pollutant.

To separate personal exposure into indoor- and outdoor-generated exposures, we followed a different approach for the 15-min intervals that participants were at home or not. When at home, exposure to outdoor sources was defined as the proportion of ambient pollutant infiltrating into the home using home- and month-specific infiltration efficiencies. Exposure to indoor sources was calculated as the difference between total measured personal exposure and exposure from outdoor sources. When outdoors or other microenvironments, we assumed no other indoor sources and personal exposure from outdoor sources was equal to the measured personal exposure. COPD patients spent less than 1% of their time in other indoor microenvironments, therefore the indoor sources in these locations would contribute minorly to their daily exposures.

Below, we present example plots for the separation of indoor- and outdoor-generated air pollutants for one participant from our dataset:

Figure S1 – Example of exposure separation for one participant in our study. The concentrations are daily means expressed in µg/m³ for $PM_{2.5}$, ppb for NO_2 and ppm for CO. PeIG = personal exposure to indoor generated pollution (in red), PeOG = personal exposure to outdoor generated pollution (in green), PeT = total personal exposure (in blue).

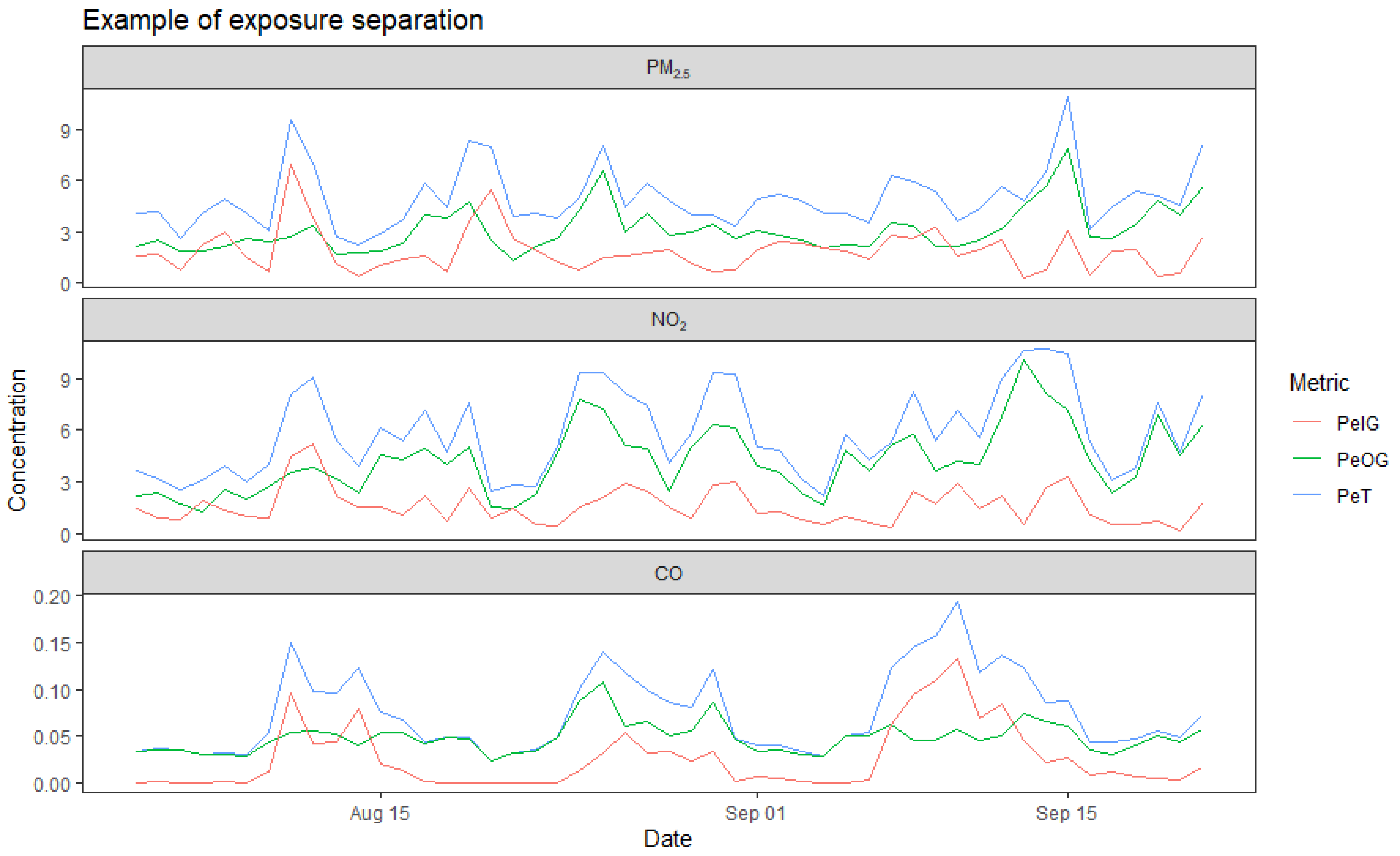

Correlation tables: Tables S1 and S2 below present median (interquartile range) within-person Pearson correlation coefficients between the different exposure metrics used in our analysis for each pollutant separately, and also between the multiple pollutants for each exposure metric separately.

Table S1 – Median (IQR) within-person Pearson correlation coefficients between the different exposure metrics by each pollutant separately. PeIG = personal exposure to indoor generated pollution, PeOG = personal exposure to outdoor generated pollution, PeT = total personal exposure.

**Median within-person Pearson correlation coefficient (IQR)**	**PeT**	**PeIG**	**PeOG**	**Ambient**
		**$PM_{2.5}$**		
**PeT**	1			
**PeIG**	0.86 (0.71 – 0.94)	1		
**PeOG**	0.51 (0.39 – 0.70)	0.03 (-0.06 – 0.21)	1	
**Ambient**	0.39 (0.21 – 0.62)	0.04 (-0.06 – 0.19)	0.84 (0.61 – 0.91)	1
		**NO_2**		
**PeT**	1			
**PeIG**	0.73 (0.55 – 0.87)	1		
**PeOG**	0.64 (0.48 – 0.76)	-0.11 (-0.21 – 0.10)	1	
**Ambient**	0.32 (0.16 – 0.52)	-0.19 (-0.41 – 0.05)	0.76 (0.59 – 0.87)	1
		**CO**		
**PeT**	1			
**PeIG**	0.86 (0.78 – 0.92)	1		
**PeOG**	0.58 (0.48 – 0.77)	0.11 (0.02 – 0.25)	1	
**Ambient**	0.34 (0.12 – 0.55)	0.02 (-0.17 – 0.15)	0.73 (0.54 – 0.87)	1
		**O_3**		
**PeT**	1			
**PeIG**	0.72 (0.64 – 0.86)	1		
**PeOG**	0.73 (0.62 – 0.84)	0.24 (0.04 – 0.40)	1	
**Ambient**	0.35 (0.13 – 0.52)	0.04 (-0.23 – 0.26)	0.59 (0.35 – 0.75)	1

Table S2 - Median (IQR) within-person Pearson correlation coefficients between the different pollutants by exposure metric separately. PeIG = personal exposure to indoor generated pollution, PeOG = personal exposure to outdoor generated pollution, PeT = total personal exposure.

Median within-person Pearson correlation coefficient (IQR)	$PM_{2.5}$	NO_2	CO	O_3
**Total Personal Exposure (PeT)**				
**$PM_{2.5}$**	1			
**NO_2**	0.20 (0.08 – 0.28)	1		
**CO**	0.27 (0.14 – 0.41)	0.35 (0.16 – 0.54)	1	
**O_3**	-0.08 (-0.19 – 0.03)	-0.25 (-0.44 – -0.04)	-0.27 (-0.36 – -0.08)	1
**Personal Exposure generated indoors (PeIG)**				
**$PM_{2.5}$**	1			
**NO_2**	0.13 (0.03 – 0.26)	1		
**CO**	0.20 (0.10 – 0.33)	0.27 (0.06 – 0.51)	1	
**O_3**	-0.06 (-0.13 – 0.05)	-0.24 (-0.37 – -0.07)	-0.12 (-0.22 – -0.03)	1
**Personal Exposure generated outdoors (PeOG)**				
**$PM_{2.5}$**	1			
**NO_2**	0.40 (0.26 – 0.53)	1		
**CO**	0.49 (0.33 – 0.64)	0.50 (0.35 – 0.62)	1	
**O_3**	-0.08 (-0.29 – 0.09)	-0.19 (-0.48 – 0.01)	-0.21 (-0.43 – -0.02)	1
**Ambient concentrations**				
**$PM_{2.5}$**	1			
**NO_2**	0.64 (0.57 – 0.72)	1		
**CO**	0.75 (0.68 – 0.85)	0.71 (0.64 – 0.79)	1	
**O_3**	-0.28 (-0.55 – 0.02)	-0.38 (-0.66 – 0.08)	-0.45 (-0.60 – -0.04)	1

Epidemiological analysis:

Tables S3, S4 and S5 present epidemiological estimates as part of the sensitivity analysis of our study, including the two-exposure models, estimates for up to 3-days lagged exposures, potential effect modification for those that stayed all day at home compared to those that went outside of their home, and, finally, our epidemiological estimates per 1 unit increase in the exposures, instead of per IQR increase.

Table S3 - Two-exposure models for the associations between personal exposure to air pollutants and exacerbation, adjusting either indoor-generated for outdoor-generated exposures and vice versa, or for the same exposure variable of another pollutant. In the diagonal are the unadjusted, single-exposure estimates (in squares) and in bold the statistically significant ORs. All estimates are per interquartile range (IQR) and adjusted for age, sex, COPD severity, Index of Multiple Deprivation (IMD) rank, inhaled corticosteroids (ICS) medication use, temperature and time. PeIG = personal exposure to indoor generated pollution, PeOG = personal exposure to outdoor generated pollution, PeT = total personal exposure.

**Exposure**	**PeIG $PM_{2.5}$ adjusted**	**PeOG $PM_{2.5}$ adjusted**	**PeIG NO_2 adjusted**	**PeOG NO_2 adjusted**	**PeIG CO adjusted**	**PeOG CO adjusted**	**PeIG adjusted**	**PeOG O_3 adjusted**
**PeIG $PM_{2.5}$**	1.006 (0.976, 1.037)	1.005 (0.975, 1.035)	1.003 (0.973, 1.034)	-	0.999 (0.969, 1.030)	-	1.006 (0.976, 1.037)	-
**PeOG $PM_{2.5}$**	0.989 (0.939, 1.041)	0.988 (0.939, 1.041)	-	0.965 (0.912, 1.021)	-	0.975 (0.922, 1.031)	-	0.982 (0.932, 1.035)
**PeIG NO_2**	**1.203 (1.125, 1.287)**	-	**1.194 (1.117, 1.276)**	**1.206 (1.128, 1.290)**	**1.181 (1.102, 1.265)**	-	**1.211 (1.133, 1.295)**	-
**PeOG NO_2**	-	**1.135 (1.057, 1.218)**	**1.134 (1.062, 1.212)**	**1.121 (1.049, 1.198)**	-	**1.114 (1.041, 1.191)**	-	**1.108 (1.037, 1.184)**
**PeIG CO**	**1.063 (1.005, 1.125)**	-	1.025 (0.967, 1.087)	-	**1.065 (1.008, 1.125)**	**1.059 (1.002, 1.120)**	**1.074 (1.016, 1.135)**	-
**PeOG CO**	-	1.034 (0.996, 1.072)	-	1.016 (0.984, 1.049)	1.022 (0.990, 1.055)	1.027 (0.994, 1.060)	-	1.015 (0.984, 1.047)
**PeIG O_3**	**1.085 (1.016, 1.158)**	-	**1.120 (1.050, 1.194)**	-	**1.093 (1.025, 1.165)**	-	**1.092 (1.024, 1.164)**	**1.117 (1.047, 1.192)**
**PeOG O_3**	-	**0.811 (0.741, 0.889)**	-	**0.849 (0.777, 0.927)**	-	**0.842 (0.770, 0.920)**	**0.815 (0.745, 0.892)**	**0.834 (0.764, 0.911)**

Table S4 – Odds ratios (OR) with 95% CI for the occurrence of exacerbation per interquartile range (IQR) increase on the same (Lag 0) or previous (Lag 1, Lag 2, Lag 3) days or the average of the same and three previous days (Lag 0-3) for each pollutant and exposure variable or for those that spent all day at home or not. Random intercept models adjusted for age, sex, COPD severity, Index of Multiple Deprivation (IMD) rank, inhaled corticosteroids (ICS) medication use, temperature and time. In bold are the statistically significant estimates. PeIG = personal exposure to indoor generated pollution, PeOG = personal exposure to outdoor generated pollution, PeT = total personal exposure.

Pollutant	Exposure Variable (IQR)	OR per IQR increase (95% CI)						
		Lag 0	Lag 1	Lag 2	Lag 3	Lag 0-3	All-day at home	Not all-day at home
PM$_{2.5}$	PeT (8.7 µg/m^3)	0.999 (0.957, 1.043)	1.01096 (0.968, 1.056)	1.023 (0.979, 1.069)	1.033 (0.988, 1.080)	1.044 (0.978, 1.115)	0.978 (0.932, 1.027)	1.051 (0.976, 1.130)
	PeIG (5.9 µg/m^3)	1.005 (0.975, 1.036)	1.016 (0.985, 1.049)	1.026 (0.994, 1.059)	**1.034 (1.001, 1.068)**	**1.057 (1.007, 1.111)**	0.991 (0.957, 1.025)	1.035 (0.975, 1.099)
	PeOG (3.8 µg/m^3)	0.988 (0.939, 1.041)	0.976 (0.924, 1.031)	0.977 (0.925, 1.033)	0.971 (0.918, 1.028)	0.962 (0.902, 1.026)	0.991 (0.901, 1.091)	1.003 (0.949, 1.059)
	Ambient (8.7 µg/m^3)	1.015 (0.962, 1.072)	1.005 (0.951, 1.062)	1.000 (0.946, 1.057)	0.981 (0.927, 1.039)	1.004 (0.940, 1.072)	0.972 (0.903, 1.045)	1.067 (0.989, 1.151)
NO$_2$	PeT (5.5 ppb)	**1.328 (1.216, 1.451)**	**1.340 (1.225, 1.466)**	**1.345 (1.229, 1.472)**	**1.342 (1.226, 1.470)**	**1.513 (1.358, 1.685)**	**1.234 (1.098, 1.387)**	**1.440 (1.281, 1.619)**
	PeIG (3.5 ppb)	**1.194 (1.117, 1.276)**	**1.198 (1.119, 1.282)**	**1.209 (1.129, 1.295)**	**1.213 (1.132, 1.300)**	**1.366 (1.250, 1.492)**	**1.161 (1.068, 1.263)**	**1.213 (1.101, 1.336)**
	PeOG (2.8 ppb)	**1.121 (1.049, 1.198)**	**1.128 (1.054, 1.206)**	**1.131 (1.056, 1.211)**	**1.128 (1.053, 1.208)**	**1.187 (1.095, 1.288)**	0.992 (0.874, 1.126)	**1.218 (1.120, 1.324)**
	Ambient (11.1 ppb)	**1.103 (1.011, 1.202)**	**1.116 (1.021, 1.219)**	**1.117 (1.021, 1.222)**	1.081 (0.987, 1.183)	**1.178 (1.057, 1.313)**	0.976 (0.871, 1.094)	**1.284 (1.139, 1.448)**
CO	PeT (0.08 ppm)	**1.051 (1.006, 1.098)**	**1.088 (1.027, 1.152)**	**1.073 (1.016, 1.132)**	**1.066 (1.008, 1.128)**	**1.085 (1.022, 1.151)**	1.024 (0.967, 1.083)	**1.087 (1.015, 1.165)**
	PeIG (0.06 ppm)	**1.065 (1.008, 1.125)**	**1.092 (1.032, 1.155)**	**1.082 (1.022, 1.146)**	**1.090 (1.029, 1.155)**	**1.163 (1.073, 1.260)**	1.042 (0.974, 1.115)	**1.087 (0.997, 1.184)**
	PeOG (0.03 ppm)	1.027 (0.994, 1.060)	**1.065 (1.014, 1.119)**	**1.114 (1.046, 1.187)**	1.006 (0.974, 1.039)	1.018 (0.987, 1.050)	1.013 (0.936, 1.097)	1.035 (0.996, 1.076)
	Ambient (0.09 ppm)	1.012 (0.964, 1.063)	1.004 (0.955, 1.056)	0.982 (0.932, 1.035)	0.958 (0.908, 1.012)	0.978 (0.919, 1.041)	0.957 (0.895, 1.023)	1.075 (1.007, 1.147)
O$_3$	PeT (4.8 ppb)	0.998 (0.913, 1.091)	0.960 (0.877, 1.051)	0.958 (0.875, 1.048)	0.949 (0.868, 1.039)	0.926 (0.826, 1.038)	1.002 (0.894, 1.122)	1.007 (0.896, 1.131)
	PeIG (2.7 ppb)	**1.092 (1.024, 1.164)**	**1.072 (1.003, 1.146)**	1.065 (0.996, 1.139)	1.052 (0.984, 1.125)	1.121 (1.027, 1.223)	1.023 (0.942, 1.110)	1.182 (1.075, 1.300)
	PeOG (3.3 ppb)	**0.834 (0.764, 0.911)**	0.824 (0.753, 0.902)	0.890 (0.788, 1.004)	0.915 (0.812, 1.031)	0.730 (0.653, 0.815)	0.848 (0.729, 0.986)	0.854 (0.773, 0.944)
	Ambient (15.4 ppb)	**1.107 (1.011, 1.213)**	1.082 (0.986, 1.188)	0.890 (0.778, 1.019)	.873 (0.762, 1.000)	**1.132 (1.013, 1.265)**	**1.201 (1.069, 1.349)**	1.013 (0.891, 1.152)

Table S5 – Odds ratio (OR) with 95% CI for the occurrence of respiratory symptoms and average change in peak expiratory flow (PEF) associated with 1 unit increase on the same day (lag0) for each air pollutant and exposure variable. Random intercept models adjusted for age, sex, COPD severity, Index of Multiple Deprivation (IMD) rank, inhaled corticosteroids (ICS) medication use, temperature and time. For CO, the exposure increment is 0.1 ppm. PeIG = personal exposure to indoor generated pollution, PeOG = personal exposure to outdoor generated pollution, PeT = total personal exposure.

Pollutant	Exposure Variable	OR (95% CI) for symptom occurrence per 1 unit increase in the exposure*						Average change in PEF (L/min) (95% CI)
		Exacerbation	Breathlessness	Cough	Sleep disturbance	Sputum	Wheeze	
$PM_{2.5}$	PeT	1.000 (0.995, 1.005)	0.998 (0.993, 1.003)	0.998 (0.993, 1.004)	0.994 (0.985, 1.002)	1.006 (0.998, 1.014)	0.997 (0.991, 1.004)	**-0.06 (-0.10, -0.03)**
	PeIG	1.001 (0.996, 1.006)	0.996 (0.990, 1.002)	0.999 (0.993, 1.005)	0.986 (0.975, 0.998)	**1.011 (1.003, 1.019)**	1.000 (0.993, 1.007)	**-0.05 (-0.09, -0.02)**
	PeOG	0.997 (0.984, 1.011)	1.006 (0.996, 1.016)	1.001 (0.986, 1.018)	1.004 (0.993, 1.015)	0.979 (0.956, 1.002)	0.978 (0.954, 1.002)	-0.04 (-0.10, 0.02)
	Ambient	1.002 (0.996, 1.008)	1.001 (0.994, 1.007)	1.006 (0.999, 1.014)	0.994 (0.990, 1.006)	0.994 (0.986, 1.003)	1.005 (0.995, 1.015)	-0.01 (-0.05, 0.03)
NO_2	PeT	**1.053 (1.036, 1.070)**	1.002 (0.986, 1.019)	**1.063 (1.044, 1.082)**	1.014 (0.995, 1.033)	**1.050 (1.029, 1.072)**	**1.032 (1.004, 1.061)**	**0.11 (0.01, 0.20)**
	PeIG	**1.052 (1.032, 1.072)**	0.994 (0.975, 1.013)	**1.029 (1.008, 1.052)**	1.015 (0.993, 1.037)	**1.041 (1.017, 1.067)**	0.995 (0.963, 1.028)	0.04 (-0.07, 0.15)
	PeOG	**1.041 (1.017, 1.066)**	1.027 (0.996, 1.058)	**1.096 (1.068, 1.125)**	1.015 (0.985, 1.046)	**1.065 (1.036, 1.095)**	**1.097 (1.050, 1.146)**	0.13 (-0.03, 0.29)
	Ambient	**1.009 (1.001, 1.017)**	**1.010 (1.001, 1.019)**	**1.019 (1.010, 1.028)**	1.007 (0.997, 1.016)	0.997 (0.986, 1.009)	**1.024 (1.011, 1.036)**	0.02 (-0.04, 0.07)
CO*	PeT	**1.066 (1.008, 1.127)**	**1.089 (1.024, 1.159)**	**1.134 (1.058, 1.215)**	0.959 (0.878, 1.047)	**1.250 (1.136, 1.374)**	**1.125 (1.023, 1.238)**	-0.06 (-0.52, 0.39)
	PeIG	**1.116 (1.014, 1.230)**	1.061 (0.956, 1.177)	**1.169 (1.052, 1.299)**	0.939 (0.825, 1.067)	**1.305 (1.147, 1.486)**	1.030 (0.905, 1.172)	0.25 (-0.50, 0.99)
	PeOG	1.083 (0.983, 1.193)	1.115 (0.994, 1.251)	**1.117 (1.005, 1.240)**	0.939 (0.790, 1.115)	**1.278 (1.085, 1.505)**	**1.427 (1.134, 1.795)**	0.59 (-0.19, 1.37)
	Ambient	1.013 (0.961, 1.069)	1.039 (0.981, 1.101)	**1.077 (1.016, 1.142)**	0.968 (0.908, 1.031)	0.940 (0.867, 1.019)	**1.101 (1.005, 1.206)**	-0.22 (-0.60, 0.16)
O_3	PeT	1.000 (0.982, 1.018)	0.988 (0.969, 1.007)	0.974 (0.953, 0.995)	1.012 (0.990, 1.035)	0.985 (0.960, 1.011)	1.002 (0.977, 1.027)	-0.07 (-0.19, 0.04)
	PeIG	**1.034 (1.009, 1.059)**	0.989 (0.963, 1.016)	0.994 (0.966, 1.023)	1.011 (0.980, 1.043)	1.008 (0.976, 1.042)	1.012 (0.979, 1.046)	-0.08 (-0.23, 0.08)
	PeOG	**0.946 (0.921, 0.972)**	0.977 (0.952, 1.002)	0.928 (0.899, 0.957)	1.010 (0.982, 1.038)	**0.954 (0.919, 0.989)**	0.990 (0.954, 1.027)	-0.01 (-0.17, 0.15)
	Ambient	**1.007 (1.001, 1.013)**	**1.010 (1.003, 1.016)**	**1.006 (1.000, 1.013)**	1.003 (0.995, 1.010)	**1.020 (1.011, 1.028)**	0.999 (0.990, 1.007)	0.02 (-0.02, 0.06)

*For CO, results are presented per 0.1 ppm increase.

References:

Allen, Ryan, et al. "Use of real-time light scattering data to estimate the contribution of infiltrated and indoor-generated particles to indoor air." Environmental science & technology 37.16 (2003): 3484-3492.

Chatzidiakou, Lia, et al. "Automated classification of time-activity-location patterns for improved estimation of personal exposure to air pollution." Environmental Health 21.1 (2022): 1-21.

Kelly, Frank et al. “The London low emission zone baseline study; Appendix B.” Res. Rep. Health Eff. Inst. (2011):3-79.

Moore, Elizabeth, et al. "Linking e-health records, patient-reported symptoms and environmental exposure data to characterise and model COPD exacerbations: protocol for the COPE study." BMJ open 6.7 (2016): e011330

Zhang, Hanbin, et al. "Partitioning indoor-generated and outdoor-generated PM2.5 from real-time residential measurements in urban and peri-urban Beijing." Science of The Total Environment 845 (2022): 157249
